# Less invasive Achilles tendon reconstruction

**DOI:** 10.1186/1471-2474-8-100

**Published:** 2007-10-26

**Authors:** Michael R Carmont, Nicola Maffulli

**Affiliations:** 1 Department of Trauma and Orthopaedics, University Hospital of North Staffordshire, Keele University School of Medicine, Stoke on Trent, ST7 4QG UK

## Abstract

**Background:**

The optimal management of chronic ruptures of the Achilles tendon is surgical reconstruction. Reconstruction of the Achilles tendon using peroneus brevis has been widely reported. Classically, these procedures involve relatively long surgical wounds in a relatively hypovascular area which is susceptible to wound breakdown.

**Results:**

We describe our current method of peroneus brevis reconstruction for the Achilles tendon using two para-midline incisions.

**Conclusion:**

This technique allows reconstruction of the Achilles tendon using peroneus brevis preserving skin integrity over the site most prone to wound breakdown, and can be especially used to reconstruct the Achilles tendon in the presence of previous surgery.

## Background

Acute Achilles tendons ruptures may be managed either operatively or non-operatively. However, generally 6 weeks following a rupture a direct repair opposing the tendon ends becomes increasingly difficult. Over time, scar tissue forms, the muscles atrophy with disuse, and the tendon ends weaken. Chronic and neglected Achilles tendon ruptures are debilitating: their optimal management is surgical [1]. Operative procedures for reconstruction of the Achilles tendon include flap tissue turn down using one [2-4] and two flaps [5], local tendon transfer [6-9], and autologous hamstring tendon harvesting [10]. All of these techniques use a single longitudinal incision for exposure. Following these procedures, complications, especially wound breakdown and infection (9%), are not infrequent, are probably related to the paucity of the soft tissue vascularity, and may require plastic surgical procedures to cover significant soft tissue defects [11-14].

We previously described our open technique to allow full exposure for late reconstruction of chronic Achilles tendon tears using peroneus brevis [9]. We describe our current method, using less invasive surgery than an open reconstruction. Our technique uses two para-midline incisions preserving skin integrity over the site most prone to wound breakdown.

## Results

The patient is positioned prone with a calf tourniquet. Skin preparation is performed in the usual fashion, and sterile drapes are applied. Pre-operative anatomical markings include the palpable tendon defect, both malleoli, and the base of the fifth metatarsal.

**Figure 1:**
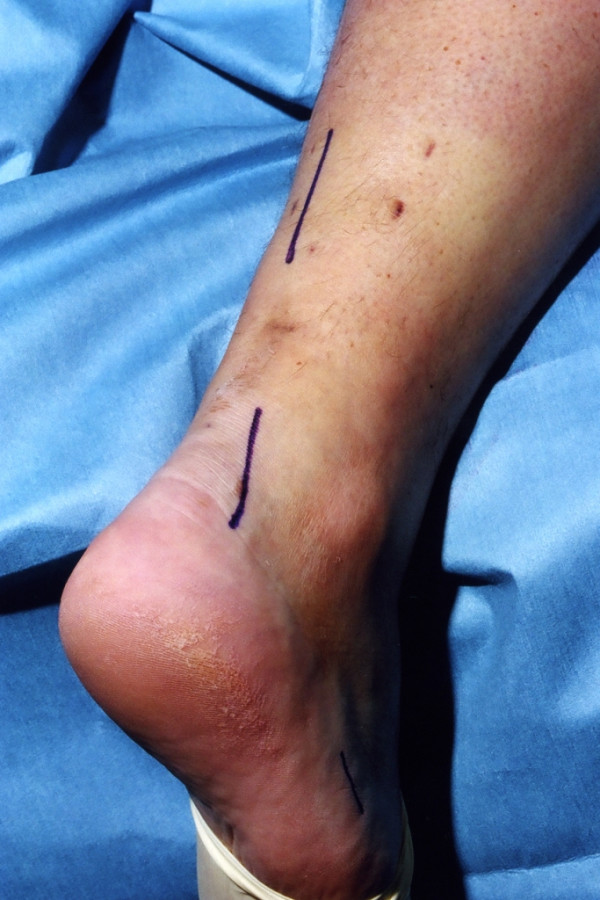
Preoperative skin markings of the 3 incisions: a 5 cm proximal longitudinal incision commencing 2 cm proximal and medial to the tendon defect, a 4 cm distal longitudinal incision commencing 2 cm distal and lateral to the tendon defect, and a 2 cm longitudinal incision proximal just superior to the tubercle of the base of the fifth metatarsal.

Three skin incisions are made (Figure 1), and accurate haemostasis by ligation of the larger veins and diathermy of the smaller ones is performed. The first incision is a 5 cm longitudinal incision, made 2 cm proximal and just medial to the palpable end of the residual tendon. The second incision is 3 cm long and is also longitudinal but is 2 cm distal and lateral to the distal end of the tendon rupture. Care is taken to prevent damage to the sural nerve by making this incision as close as possible to the anterior aspect of the lateral border of the Achilles tendon to avoid the nerve. At the level of the Achilles tendon insertion, the sural nerve is 18.8 mm lateral to the tendon but, as it progresses proximally, the nerve gradually traverses medially crossing the lateral border of the tendon 9.8 cm proximal to the calcaneum [15]. Thus, the second incision avoids the sural nerve by being placed on the lateral side of the Achilles tendon but medial to the nerve. The third incision is a 2 cm longitudinal incision at the base of the fifth metatarsal.

**Figure 2:**
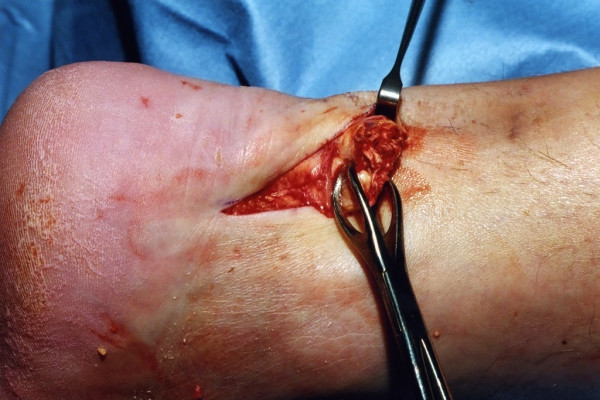
The distal portion of the Achilles tendon is mobilised to the calcaneum.

**Figure 3:**
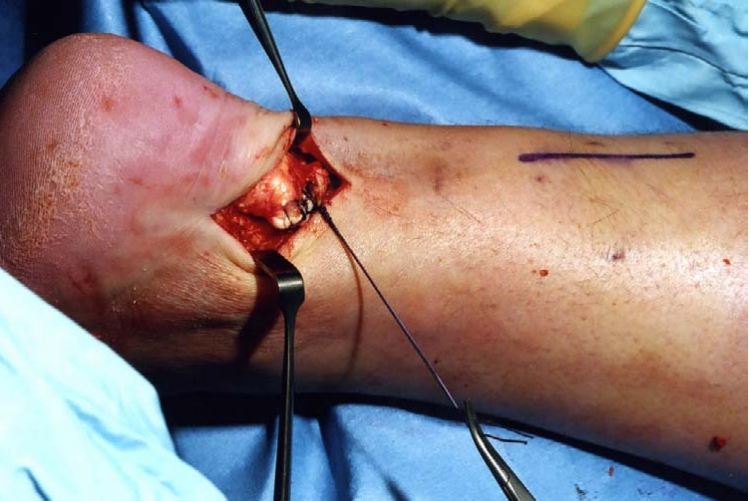
The distal end of the ruptured tendon is resected back to healthy tendon tissue, and the end is secured with a Vicryl locking suture.

**Figure 4:**
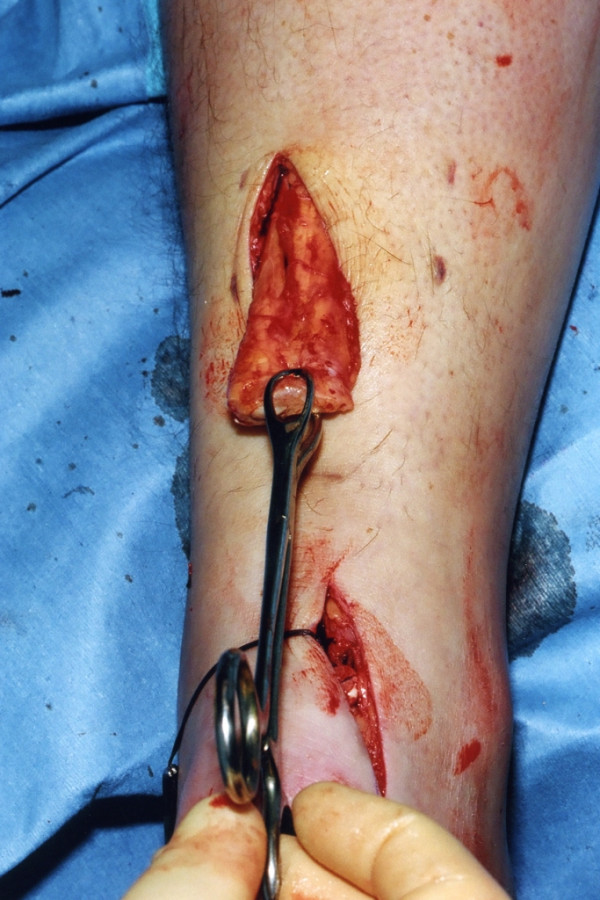
The proximal portion of the Achilles tendon is mobilised through the proximal incision.

The distal Achilles tendon stump is mobilised, freeing it of all the peritendinous adhesions, particularly on the lateral aspect (Figure 2). This allows access to the base of the lateral aspect of the distal tendon close to it insertion. It should be possible to palpate the medial tubercle of the calcaneum (Figure 3). The ruptured tendon end is then resected back to healthy tendon, and a Number 1 Vicryl (Ethicon, Edinburgh) locking suture is run along the free tendon edge to prevent separation of the bundles (Figure 4).

**Figure 5:**
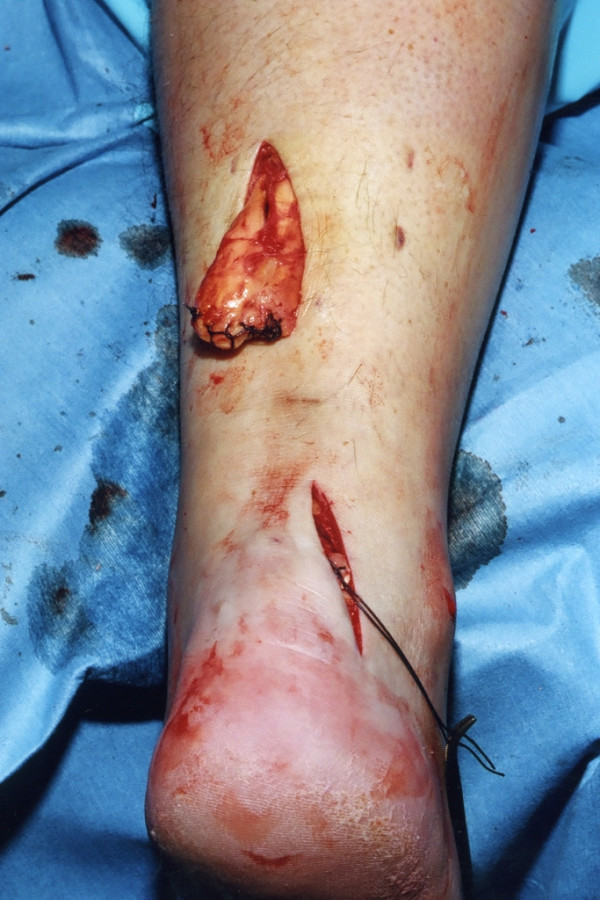
The proximal portion of the Achilles tendon is resected back to healthy tendon, and the tendon end is secured with a Vicryl locking suture.

The proximal tendon is then mobilised from the proximal wound, any adhesions are divided, and further soft tissue release anterior to the soleus and gastrocnemius allows maximal excursion, minimising the gap between the two tendon stumps. A Vicryl locking suture is run along the free tendon edge to allow adequate exposure and to prevent separation of the bundles (Figure 5).

**Figure 6:**
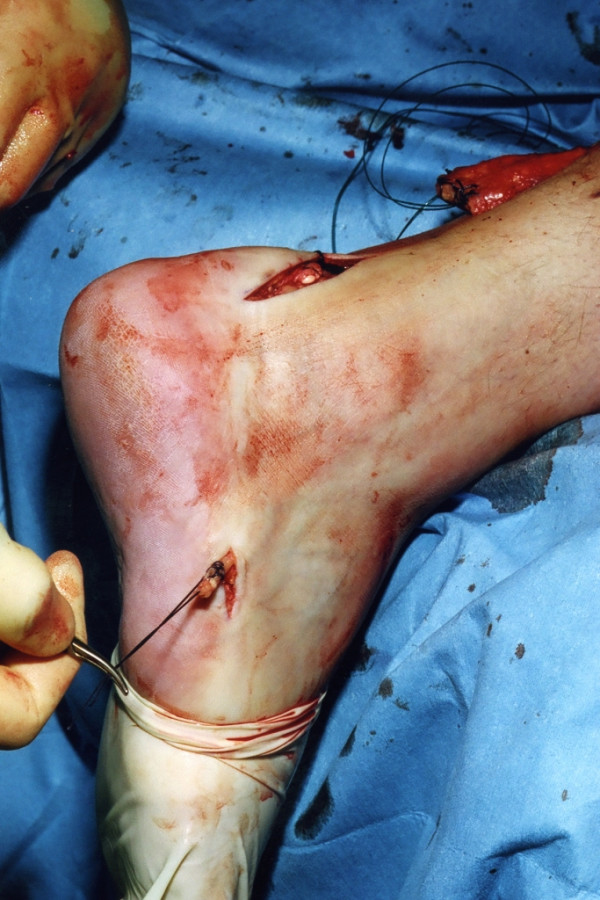
Through the incision over the lateral aspect of the foot, the tendon of peroneus brevis is identified, held with a locking suture, released from the base of the fifth metatarsal, and mobilised.

**Figure 7:**
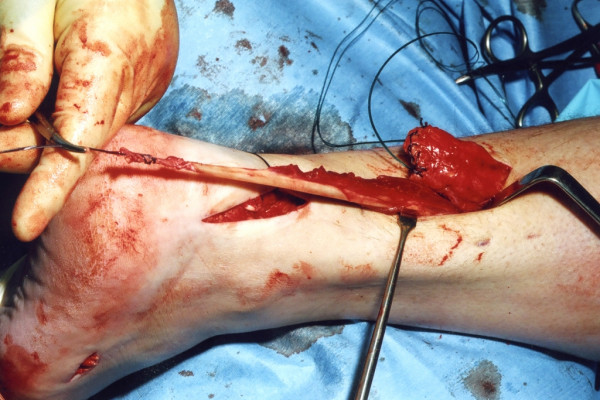
The tendon of peroneus brevis is then passed out of the proximal wound and mobilised to allow increased excursion.

The tendon of peroneus brevis is harvested. The tendon is identified through the incision on the lateral border of the foot at its insertion at the base of the fifth metatarsal. The tendon is exposed, and a No.1 Vicryl locking suture is applied to the tendon end before release from the metatarsal base (Figure 6). The tendon of peroneus brevis is identified at the base of the distal incision of the Achilles tendon following incision of the deep fascia overlying the peroneal muscles compartment. The tendon of peroneus brevis is then withdrawn through the distal wound. This may take significant force, as there may be tendinous strands between the two peroneal tendons distally. The muscular portion of peroneus brevis is then mobilised proximally to allow increased excursion of the tendon of peroneus brevis (Figure 7).

**Figure 8:**
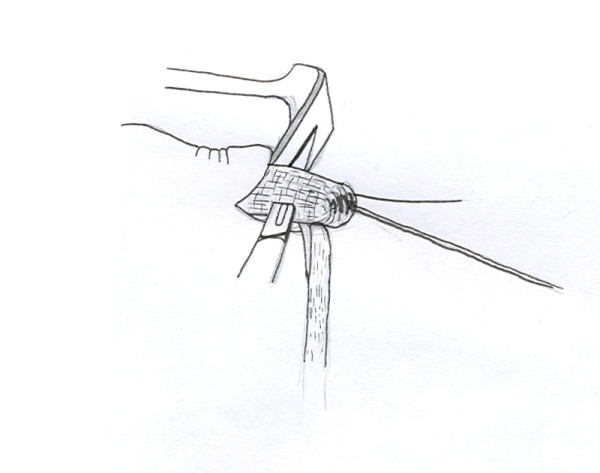
A No 11 blade is used to make a coronal tenotomy in the distal stump of the Achilles tendon.

**Figure 9:**
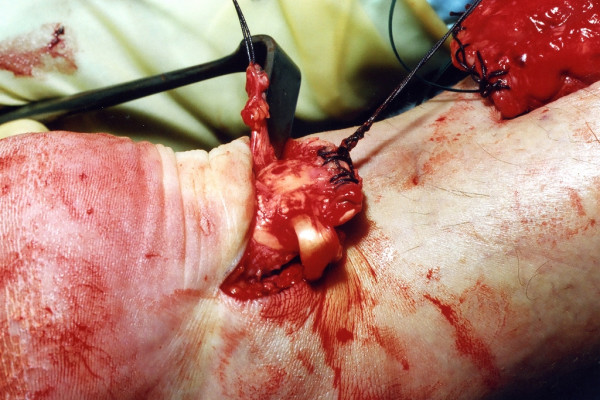
The peroneus brevis tendon is then passed through the tenotomy in the Achilles tendon.

**Figure 10:**
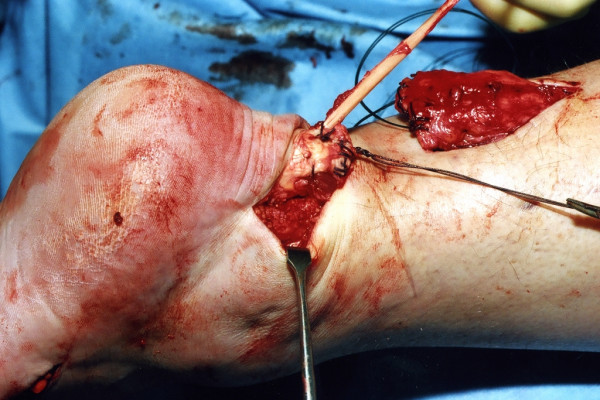
With the ankle in full equinus, the peroneus brevis tendon is sutured to the distal stump of the Achilles tendon on both sides of the stump.

**Figure 11:**
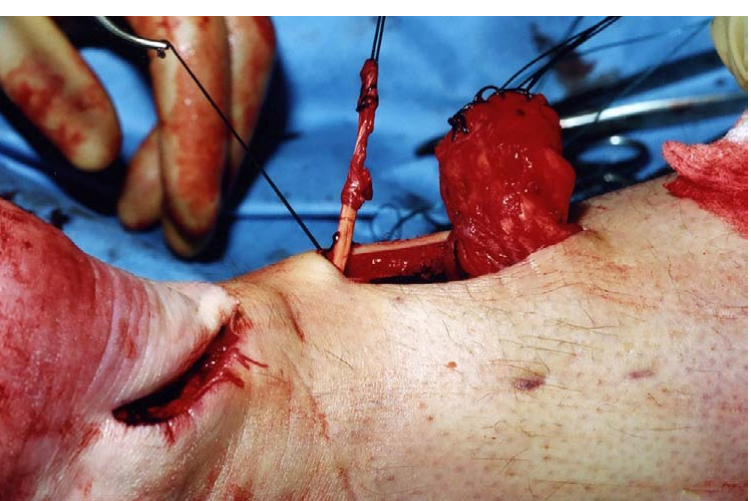
The peroneus brevis tendon is then passed under the skin bridge into the proximal wound.

**Figure 12:**
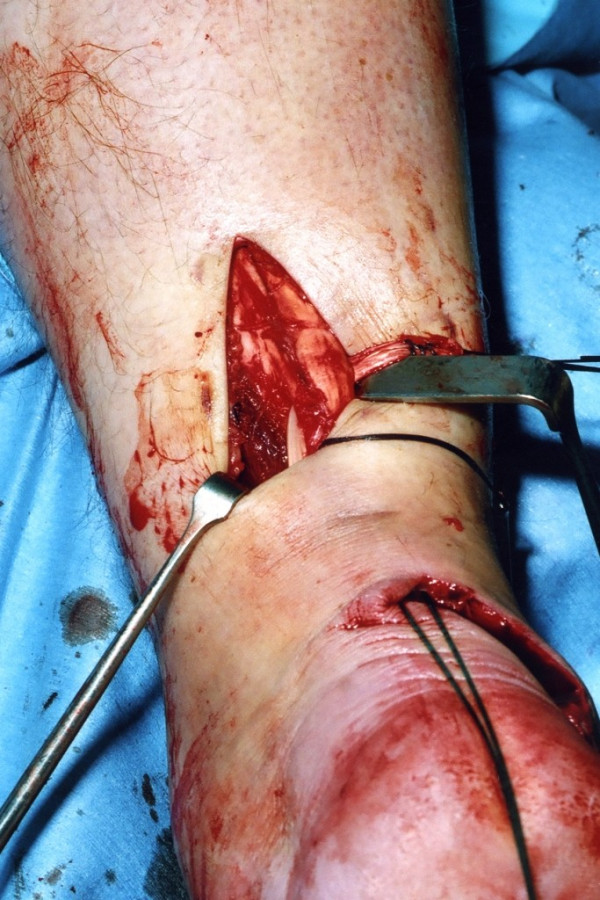
A second transverse tenotomy is made in the proximal stump of the Achilles tendon, the tendon of peroneus brevis is passed through this tenotomy, and pulled distally.

**Figure 13:**
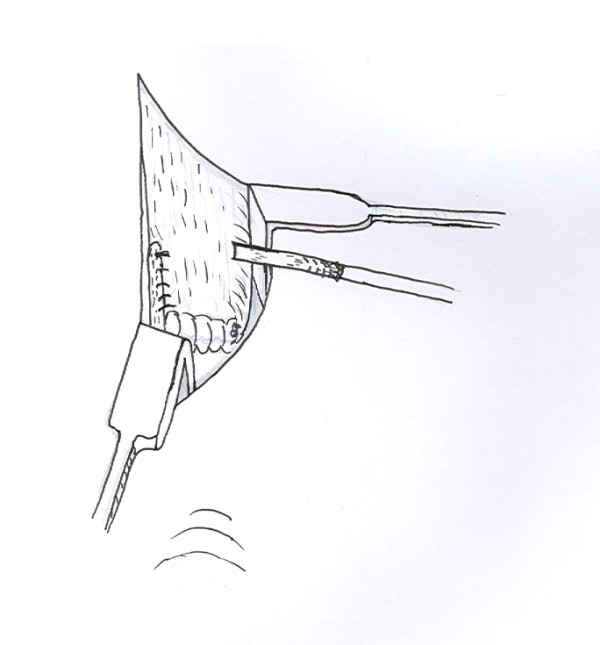
A locking suture is used to secure the graft tendon to both sides of the proximal portion of the Achilles tendon and back onto itself.

A longitudinal tenotomy parallel to the tendon fibres is made through both stumps of the tendon (Figure 8). A clip is used to develop the plane, from lateral to medial, in the distal stump of the Achilles tendon, and the peroneus brevis graft is passed through the tenotomy (Figure 9). With the ankle in maximal plantar flexion, a No.1 Vicryl suture is used to suture the peroneus brevis to both sides of the distal stump (Figure 10). The tendon of peroneus brevis is then passed beneath the intact skin bridge into the proximal incision (Figure 11), and passed from medial to lateral through a transverse tenotomy in the proximal stump (Figure 12), and further secured with No 1 Vicryl (Figure 13). Finally, the tendon of peroneus brevis is sutured back onto itself on the lateral side of the proximal incision. The reconstruction may be further augmented using a Maxon (Tyco Health Care, Norwalk, CT) suture.

**Figure 14:**
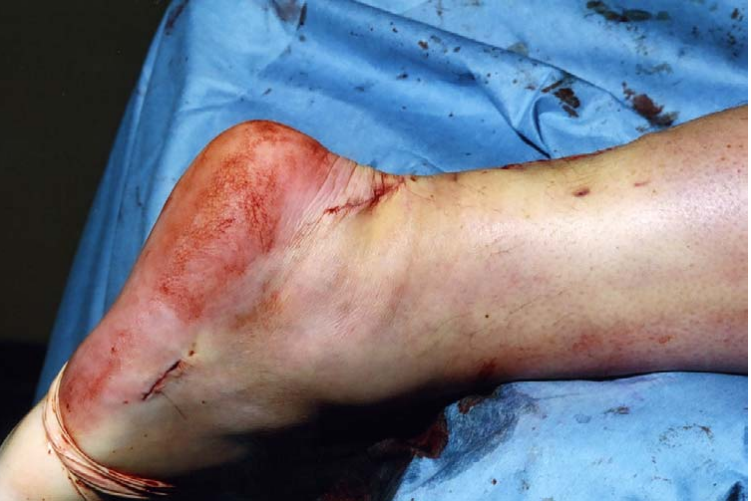
The skin wounds are closed with subcutaneous Vicryl, subcuticular Biosyn, and Steri-strips.

The wounds are closed with 2,0 Vicryl, 3,0 Biosyn (Tyco Health Care, Norwalk, CT) and Steri-strips (3 M Health Care, St Paul, MN), taking care to avoid the risk of post operative haematoma and minimise wound breakdown (Figure 14). A previously prepared removable scotch cast support with Velcro straps is applied.

Post operatively, patients are allowed to weight bear as comfort allows with the use of elbow crutches. It would be unusual for a patient to weight bear fully at this stage. After 2 weeks, the back shell is removed, and physiotherapy is commenced with the front shell *in situ *preventing dorsiflexion of the ankle, focusing on proprioception, plantar-flexion of the ankle, inversion and eversion. During this period of rehabilitation the patient is permitted to weight bear as comfort allows with the front shell *in situ *although full weight bearing rarely occurs on account of balance difficulties and patients usually still require the assistance of a single elbow crutch as this stage. The front shell may be finally removed after 6 weeks. We do not use a heel raise after removal of the cast, and patients normally regain a plantigrade ankle over a couple of weeks.

## Discussion

The use of peroneus brevis for reconstruction of chronic Achilles tendon tears is well established [16]. Achilles tendon reconstruction with peroneus brevis is advantageous in patients involved in sports [16], leaving minimal or no objective plantar flexion weakness following the procedure [17], and minimal re-rupture rates [18]. Peroneus brevis fulfils many of the essential criteria for tendon transfer [19]. The tendon has an acceptable strength of 116.2 N/mm, cross sectional area of 19.5 cm^2 ^and an elastic modulus of 149.7 N/mm^2^, compared to an ultimate tensile load of 1724 N [20-22], has similar line of pull, is in phase, has adequate excursion, and is expendable. It is also easily identified distally inserting into the tubercle of the base of the fifth metatarsal, whereas at this level the tendon of peroneus longus has already passed within its groove on the plantar aspect of the cuboid [23].

Reconstruction techniques include passing the tendon through a tunnel drilled through the calcaneus [24] and a tenotomy in the proximal Achilles tendon stump [25], and through tenotomies in the distal and the proximal tendon stumps as an open procedure [9,24]. These techniques use relatively long longitudinal incisions, and wound breakdown may occur. In our hands the open technique had a 9% superficial infection rate [17]. In many instances, reconstructive procedures for the Achilles tendon have incorporated plastic surgical flap procedures to facilitate skin closure [11,13,14].

Following surgery, the ankle is kept in equinus to prevent disruption of the reconstruction. Vascularity of the soft tissues is maximal at 20° of plantar flexion, and at 40° of plantar flexion the blood supply of the skin is reduced by 49% [26]. Therefore, the tightness of the repair may influence wound healing.

In patients with chronic ruptures, the skin over the gap retracts over several weeks, and remains so until the operation. In open surgery, this skin is incised, and is then stretched out in a relatively acute fashion to accommodate the reconstructed tendon. Therefore, following the reconstruction, the skin over the gap may well be stretched so much that vascular supply is impaired [9].

The reconstructed gastro-soleus Achilles tendon complex will stretch with increased loading and range of movement exercises during rehabilitation [27].

Preservation of skin cover during reconstruction procedures is clearly an advantage, as the skin is not injured by the operation, and protects the reconstruction beneath. As with all surgery performed through less invasive incisions, this procedure is technically demanding. Careful incision placement is required together with skin retraction to allow visualisation of the tendon ends and to permit the reconstruction. The technique is designed to preserve skin cover of the reconstruction site, and, although reconstruction is always risky, it may extend the indications for surgery in groups prone to wound complications such as vasculopaths and diabetics.

## Conclusion

This technique allows reconstruction of the Achilles tendon using peroneus brevis preserving skin integrity, and can be especially used to reconstruct the Achilles tendon in the presence of previous surgery.

## Competing interests

The author(s) declare that they have no competing interests.

## Authors' contributions

MC has performed the literature review and written the technical advance. NM is an experienced surgeon who has developed the technique and has also written and proof read the paper. All authors have read and approved the final manuscript.

## Pre-publication history

The pre-publication history for this paper can be accessed here:


